# iLIR database: A web resource for LIR motif-containing proteins in eukaryotes

**DOI:** 10.1080/15548627.2016.1207016

**Published:** 2016-08-02

**Authors:** Anne-Claire Jacomin, Siva Samavedam, Vasilis Promponas, Ioannis P. Nezis

**Affiliations:** a School of Life Sciences, University of Warwick, Coventry, UK; b Bioinformatics Research Laboratory, Department of Biological Sciences, University of Cyprus, Nicosia, Cyprus

**Keywords:** AIM, ATG8, database, LC3-interacting region motif, LIR, LIR-containing protein, LIRCP, LRS, prediction

## Abstract

Atg8-family proteins are the best-studied proteins of the core autophagic machinery. They are essential for the elongation and closure of the phagophore into a proper autophagosome. Moreover, Atg8-family proteins are associated with the phagophore from the initiation of the autophagic process to, or just prior to, the fusion between autophagosomes with lysosomes. In addition to their implication in autophagosome biogenesis, they are crucial for selective autophagy through their ability to interact with selective autophagy receptor proteins necessary for the specific targeting of substrates for autophagic degradation. In the past few years it has been revealed that Atg8-interacting proteins include not only receptors but also components of the core autophagic machinery, proteins associated with vesicles and their transport, and specific proteins that are selectively degraded by autophagy. Atg8-interacting proteins contain a short linear LC3-interacting region/LC3 recognition sequence/Atg8-interacting motif (LIR/LRS/AIM) motif which is responsible for their interaction with Atg8-family proteins. These proteins are referred to as LIR-containing proteins (LIRCPs). So far, many experimental efforts have been carried out to identify new LIRCPs, leading to the characterization of some of them in the past 10 years. Given the need for the identification of LIRCPs in various organisms, we developed the iLIR database (https://ilir.warwick.ac.uk) as a freely available web resource, listing all the putative canonical LIRCPs identified in silico in the proteomes of 8 model organisms using the iLIR server, combined with a Gene Ontology (GO) term analysis. Additionally, a curated text-mining analysis of the literature permitted us to identify novel putative LICRPs in mammals that have not previously been associated with autophagy.

## Introduction

Autophagy is a cellular catabolic process allowing for the degradation of numerous cytoplasmic components in a controlled and specific manner through the action of protein receptors that interact with Atg8/LC3/GABARAP-family proteins (hereafter refers as ‘Atg8-family proteins’).^1^

The term selective autophagy has been coined to refer to the targeted degradation of organelles (mitophagy, reticulophagy or pexophagy),^2-4^ bacteria and viruses (xenophagy),^5^ ribosomes (ribophagy),^3^ lipid droplets (lipophagy)^6^ and protein aggregates (aggrephagy).^7^ Due to the large variety of substrates, selective autophagy employs various receptors able to recognize and tether specific substrates to phagophores.

Various studies pointed out that the interaction between receptors and Atg8-family proteins is mediated by an LC3-interacting region (LIR), also known as LC3 recognition sequence (LRS) or Atg8-interacting motif (AIM).^8-15^ Thus, the presence of a LIR appears as a hallmark of the Atg8-interacting proteins.

The LIR corresponds to the shortest sequence required for the interaction with an Atg8-family protein. Previously described as the **W**xx**L** motif (where x can be any amino acid), we and others recently extended this sequence to 6 amino acids based on the multiple alignment of LIR sequences from proteins described to interact in a LIR-dependent manner with Atg8-proteins.^10,16,17^ Based on the in silico analysis of experimentally verified functional LIR motifs, we redefined the sequence of the LIR motif. The resulting consensus sequence—referred to as the xLIR motif—is (ADEFGLPRSK)(DEGMSTV)(**WFY**)(DEILQTV)(ADEFHIKLMPSTV)(**ILV**), where the residues marked in bold (positions 3 and 6) correspond to the most crucial residues for the interaction with Atg8-family proteins. An xLIR motif overlapping a region with the potential to transit from a disordered to an ordered state provides a reliable candidate for a functional binding motif.^10,17,18^

In addition to selective autophagy receptors, Atg8-family proteins can bind a variety of proteins in an LIR-dependent manner. Indeed, many LIR motif-containing proteins (LIRCPs) are required for the formation of the autophagosome,^16,19-22^ or vesicular transport,^23,24^ or they are proteins that are directly targeted to the phagophore for autophagic clearance.^25-27^

It is worth mentioning that LIR motif-independent modes of interaction with Atg8-family proteins have also been reported both in selective autophagy receptors and in other autophagy-related proteins.^28^

In this report, we describe the use of the iLIR server^17^ combined with a Gene Ontology (GO) term analysis to sort the genes from 8 model organisms (*Arabidopsis thaliana, Caenorhabditis elegans, Danio rerio, Gallus gallus, Homo sapiens, Mus musculus, Rattus norvegicus* and *Saccharomyces cerevisiae*) encoding proteins containing at least one xLIR motif inside an intrinsically disordered region. The data have been collected in the iLIR database (https://ilir.warwick.ac.uk), with the aim to provide a useful resource to researchers interested in studying the Atg8-family proteins interactome. Additionally, a curated text-mining analysis of the literature permitted us to sort human and mouse proteins known to be a part of the Atg8-family proteins interactome or to be involved in pathways linked to autophagy, and also to identify novel putative LICRPs that have not been associated with autophagy previously.

## Results and discussion

### Content of the iLIR database

The iLIR database is a web resource freely available at https://ilir.warwick.ac.uk. The website has been designed to give the user an easy way to browse available data and perform BLAST-based searches using a protein sequence of interest against part or all the sequences available in the database for proteins containing a similar xLIR motif. The website also provides hyperlinks to the UniProt database for each entry and the possibility to download the data.

**Figure 1.**
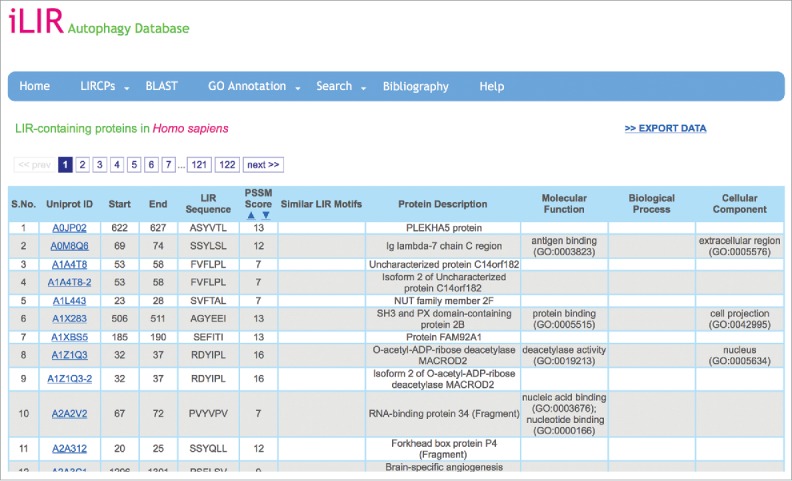
Screenshot of an iLIR database data page. In the ‘LIRCPs’ menu, the user can access the full data available in the database for each model organism. The data are arranged in a table giving various information for each entry, such as the Uniprot Accession ID and protein name, the position and sequence of the xLIR as well as the position-specific scoring matrix (PSSM) score and the similarity of other validated LIR motifs. The data can be downloaded directly.

Within the iLIR database different functionalities are organized under specific menus. The ‘LIRCPs’ menu gives access to the full list of putative LIRCPs listed in the database for the different model organisms analyzed. For a specific organism, data are presented in a table containing the following information for each entry: (i) the UniProtKB accession of the protein, (ii) the position, sequence and position-specific scoring matrix score of the xLIR,^17^ (iii) similar LIR motif in experimentally characterized LIRCPs (if any), (iv) the name of the protein, and (v) the UniProt derived GO terms associated with the molecular function, biological process and cellular component classes. The full table of data can be downloaded as an Excel file (Fig. 1).

**Figure 2.**
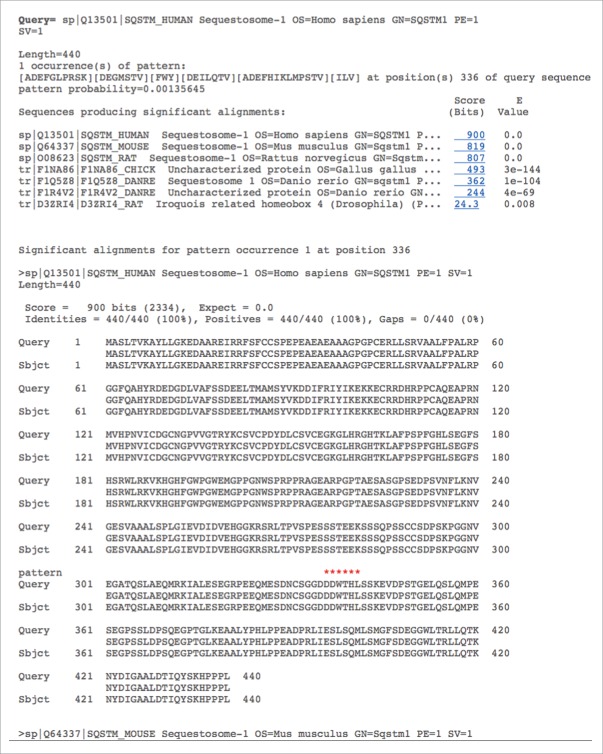
Screenshot of the iLIR database BLAST results page. Using the ‘BLAST’ menu, the user has the possibility to blast the sequence of interest against the sequences for one or all organisms available in the database in order to identify similar putative LIRCPs. The results page gives the list of similar sequences and the position of the putative LIR motif is indicated with red asterisks. The ‘sp’ and ‘tr’ preceding the FASTA header of the sequences producing a significant alignment refer to UniProtKG/Swiss-Prot (reviewed and manually annotated sequences) and UniProtKG/TrEMBL (unreviewed, automatically annotated sequences from large-scale screens), respectively.

The ‘Search’ menu offers the user to screen their sequence of interest for the presence of LIR (xLIR and WxxL) motifs using the iLIR server as described elsewhere.^17^ In addition, the user has the possibility to search in the database using specific keywords: gene name, protein description or UniProt identifier. The user may also look directly for the presence of similar proteins with the ‘BLAST’ page using PSI-BLAST.^29^ The search can be run against Swiss-Prot and TrEMBL entries from the UniProt database (a total of 276,499 FASTA sequences). The results page shows pattern positions in the query sequence and the corresponding matching positions in the subject sequences from the database along with the alignments between them. Red asterisks match the position of the conserved xLIR motif in the subject sequences. Subject sequences matched are named by their UniProtKB accession number and a link permits the redirection to the UniProtKB page for each entry (Fig. 2).

Finally, the ‘GO Annotation’ menu provides pre-computed information relative to the GO terms distribution for the LIRCPs identified for each organism. Three types of analyses are available: (i) The ‘GO Slim’ submenu directs users to a list of reduced GO terms and their abundance for each category in a specific organism. The user can sort the entries based on their counts or adjusted p-value. (ii) The ‘Distribution’ submenu directs users to a bar chart view of the GO terms distribution for each organism. (ii) The ‘Enrichment’ submenu permits the visualization of the proportion of entries for each GO term for the LIRCPs for any pair of species available in the iLIR database (Fig. S1).

### Prediction of the LIR-containing proteins (LIRCP) in the proteome of model organisms

**Figure 3.**
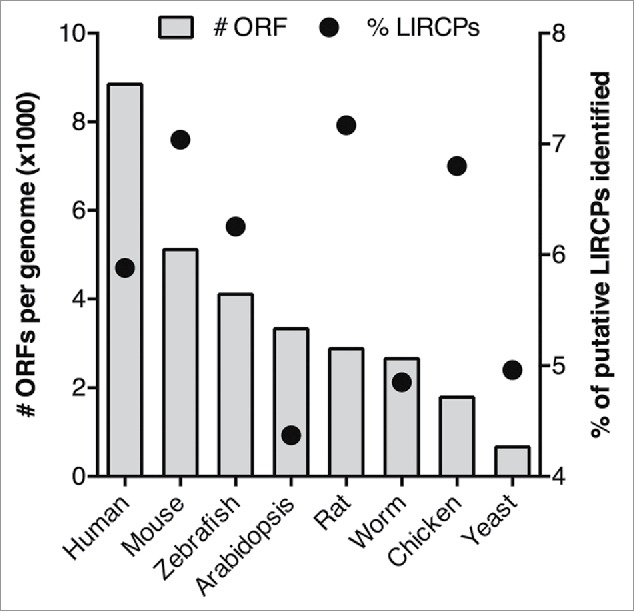
Representation of the number of ORFs (bar chart, plotted on the left axis) and percentage of putative LIRCPs identified (dot chart, plotted on the right axis) for each of the model organisms analyzed.

Using iLIR, a computational approach for predicting LC3-interaction regions in proteins,^17^ we identified putative LIRCPs from 8 model organisms (see Methods for details). We found that the proportion of putative LIRCPs varies between 4% to 7% of the total ORFs for each organism but we observed no correlation between the proportion of LIRCPs and the size of the proteome (number of ORFs) (Fig. 3 and Table 1).

**Table 1.** Summary of the numbers of putative LIRCPs and LIR identified for the 8 model organisms studied.

		# ORF	# LIRCPs	LIR motifs	% LIRCPs	Ratio #LIRs:LIRCP
Human	*Homo sapiens*	88479	5204	6087	5.88	1.17
Mouse	*Mus musculus*	51130	3599	4218	7.04	1.17
Zebrafish	*Danio rerio*	41102	2571	3007	6.26	1.17
Arabidopsis	*Arabidopsis thaliana*	33350	1458	1592	4.37	1.09
Rat	*Rattus norvegicus*	28849	2068	2425	7.17	1.17
Worm	*Caenorhabditis elegans*	26595	1290	1526	4.85	1.18
Chicken	*Gallus gallus*	17864	1215	1400	6.80	1.15
Yeast	*Saccharomyces cerevisiae*	6693	332	363	4.96	1.09

### Text-mining analysis for the identification of novel LIRCPs in mammals

In order to further investigate novel putative LIRCPs in mammals, we first concentrated on the human and mouse proteomes. Our batch analysis lead to the identification of 6087 and 4218 entries, respectively. Consecutively to the application of the statistical significance for each GO slim category for these organisms, we decided to eliminate the entries sorted as ‘non significant’ (adjusted p-value > 0.1) from the rest of the analysis. This procedure permitted us to sort a total of 1766 and 1976 entries for the human and mouse proteome, respectively, with a low to high significance level (p-adj ≤ 0.1). We made use of these significant hits for further analysis.

Previous studies have identified and described 31 proteins encoded by the human, yeast and *Arabidopsis thaliana* genomes involved in autophagy through their interaction with at least one protein belonging to the Atg8-family and containing a functional, verified LIR motif.^16,28,30^ However, the LIR motifs of a few of these proteins are not contained within an intrinsically disordered region such as human ATG4B or yeast Atg3 and Atg19.^17^ From the 31 verified LIRCPs, all 21 proteins with a LIR motif within an anchor region have been successfully identified in our computational analysis, thus validating the sorting procedure (these proteins constitute the group ‘A’ in the rest of the text) (Table S1).

**Figure 4.**
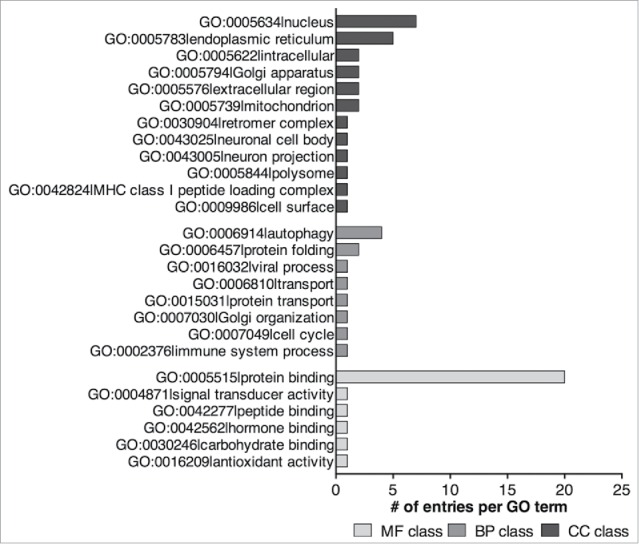
Distribution of the GO terms of the 21 human proteins listed in Kalvari et al. which have a verified xLIR in an intrinsically disordered region (see also Table S1). MF, Molecular Function; BP, Biological Process; CC, Cellular Component.

From these proteins, we extracted their associated GO slim categories for the 3 GO classes (Molecular Function, Biological Process and Cellular Component). Totally, 26 different GO terms were obtained (6 for the Molecular Function class, 8 for the Biological Process class and 12 for the Cellular Component class) (Fig. 4 and Table S1). We noticed that only 4 of these proteins have been assigned to the GO term ‘GO:0006914|autophagy’ as a Biological Process; other proteins have been assigned to GO terms that can be related to autophagy such as GO:0005739|mitochondrion, GO:0030904|retromer complex (Cellular Component), GO:0006810|transport (Biological Process), GO:0005515|protein binding and GO:0042277|peptide binding (Molecular Function). Additionally, various GO terms not directly related to autophagy have been pinpointed such as GO:0005634|nucleus, GO:0005576|extracellular region, GO:0009986|cell surface, GO:0006457|protein folding, GO:0007049|cell cycle, GO:0004871|signal transducer activity or GO:0042562|hormone binding. This suggests that many proteins whose original function is not related to autophagy might interact with Atg8-family proteins in a way that remains unknown. In order to test this assumption, we decided to screen all the putative LIRCPs with a significant adjusted p-value (sorted as previously described) for the human and mouse proteomes, which are associated with at least one of the 26 GO terms correlated with the 21 experimentaly validated human and yeast LIRCPs. Over 1,000 entries have thus been filtered. A manually curated search of these entries using PubMed, permitted us to sort 18 proteins already described to interact with an Atg8-family protein, irrespective of further evidence of a direct interaction (referred to hereafter as group ‘B’, Table S2). Three of these proteins—GPSM1/AGS3,^31,32^ NCOA4^33^ and MAPK8IP1/JIP1^34^—had been shown to interact directly (i.e., through in vitro studies) with some members of the Atg8-family. The 15 remaining proteins—PICALM,^35^ PCM1,^36^ STAT1,^37,38^ UBQLN1 and UBQLN2,^39,40^ PEG3,^41,42^ HTT,^43^ SYNPO2,^44^ UBR4,^45,46^ MAP1S,^47^ BCL10,^48^ OFD1,^36^ FNIP2,^49^ APC^50^ and CSPG4^50^—have been identified to function in complexes containing Atg8-family proteins *in cellulo* by co-immunoprecipitation and/or colocalization experiments (Table S2). In line with the functions of the LIRCPs containing experimentally verified LIR motifs (Table S1), it appears that the proteins interacting with Atg8-family members we sorted can be related to the autophagy process in various ways. Some of these Atg8-interacting proteins are selective autophagy receptors for the targeting of specific cargos (*e.g.*, NCOA4, PICALM, PCM1, STAT1),^33,35,37,38^ whereas others are degraded themselves by autophagy (*e.g.*, BCL10, OFD1).^36,48^ Yet some others are implicated in the regulation of the autophagic process (*e.g.*, GPSM1/AGS3, MAPK8IP1/JIP1, UBQLN, PEG3, HTT, SYNPO2, UBR4, MAP1S, FNIP2) (Table S2).^32,34,39-47,49^

In addition, our text-mining analysis permitted us to sort 256 supplementary entries corresponding to proteins that have been demonstrated to be involved in the regulation of autophagy, the degradation of specific substrates, or to be themselves degraded by autophagy without any evidence of interaction with Atg8-family proteins (referred to hereafter as group ‘C’, Table S3). These proteins have been described to take part in a broad range of processes related to autophagy, such as immunity (NFKBIA/IκBα,^51^ IRF1 [interferon regulatory factor 1],^52,53^ PPP1R13L/iASPP,^54^ EIF2AK2/PKR,^55^ RELA/NF-κB-p65^56,57^) or oncogenesis (BRCA1,^58,59^ MYC,^60,61^ RB1 [retinoblastoma 1],^62,63^ TSC2/Tuberin,^64^ FOXO1,^65^ XIAP^66,67^). A few posttranslational modification enzymes have also been identified, such as 2 ubiquitin ligases (HERC1^68,69^ and XIAP^66,67^), 4 kinases (PKD2/polycystin 2,^70,71^ MARK4,^72^ SIK2,^73^ CAMKK2/CaMKKβ^74^), one deacetylase (HDAC4^75^), one methyltransferase (EHMT2/G9a^76,77^) and one phosphatase (PTPN13/PTPL1^78^) (Table S3).

**Figure 5 .**
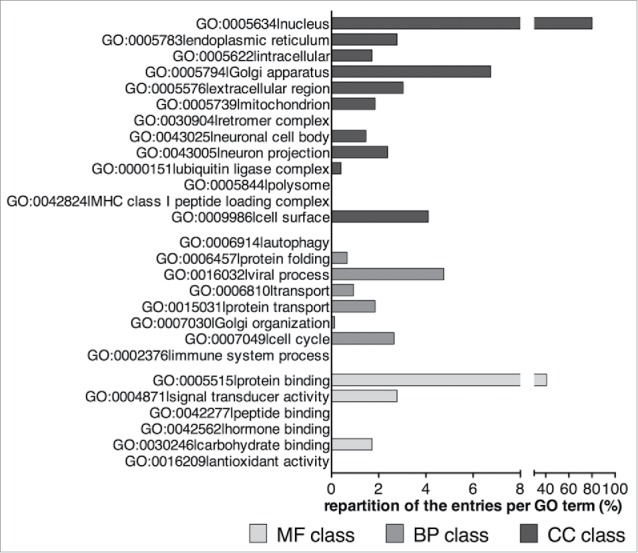
Distribution of the GO terms of the 756 human entries that have not been linked to autophagy-associated processes (see also Table S4). MF, Molecular Function; BP, Biological Process; CC, Cellular Component.

Finally, we sorted proteins that have not been shown to be linked to autophagy or associated pathways, totaling for the human proteome 756 entries sharing their GO terms with the 21 human and yeast proteins that contain experimentally verified LIR motifs in an intrinsically disordered region (refers hereafter as group ‘D’, Table S4). The most represented GO terms are GO:0005634|nucleus (80.16%) for the Cellular Component class, GO:0005515|protein binding (40.87%) for the Molecular Function class and GO:0016032|viral process (4.76%) for the Biological Process class (Fig. 5). This observation suggest that these proteins are promising candidates for further investigation.

## Conclusion

Autophagy is a vital catabolic process for the maintenance of cell and tissue homeostasis by the selective degradation and recycling of macromolecules and organelles. In recent years, great efforts have been made for the identification and characterization of new receptors for selective autophagy, leading to the discovery of the LC3-interacting region.^8-10^ Additional studies showed that LIR-containing proteins (LIRCPs) participate in a broad range of autophagic functions such as the selective targeting of cargo for degradation, the initiation and maturation of the autophagosome or vesicular transport.^79^ Given the need for the identification of novel LIRCPs, we used the iLIR server to generate the iLIR database, a comprehensive bioinformatics resource for all the putative LIRCPs identified from the proteome of 8 model organisms. Our comprehensive manual literature analysis of human and mouse proteomes shows that our database includes already experimentally validated LIRCPs and novel putative functional LIRCPs.

Of course, there are some limitations to the iLIR database. At the moment, the iLIR server is not able to predict the noncanonical LIR motifs such as the one allowing for the interaction between CALCOCO2/NDP52 and LC3C.^80^ Therefore the iLIR database cannot currently offer the list of unconventional LIRCPs.

In summary, we anticipate that the iLIR database will help autophagy researchers to test their candidates of interest, and elucidate the full set of LIRCPs in eukaryotes.

## Methods

### Proteomes of model organisms and prediction of the LIR-containing proteins (LIRCPs)

We selected 8 model organisms: *Arabidopsis thaliana, Caenorhabditis elegans, Danio rerio, Gallus gallus, Homo sapiens, Mus musculus, Rattus norvegicus* and *Saccharomyces cerevisiae*. The protein sequences encoding the complete genomes of these model organisms were obtained from the UniProt database (Uniprot.org, (2014). *UniProt*. [online] Available at: http://www.uniprot.org/ [Accessed 06 February 2014]). A stand-alone version of iLIR was employed to process the data in batch mode and predict LIRCPs based on the presence of at least one xLIR within an intrinsically disorderd region.

### Gene Ontology (GO) enrichment analysis

The GO enrichment analysis was performed by downloading the ID (identifiers) mapping data for each organism from UniProt. These data contains cross-references for a given UniProt identifier with mappings to multiple databases such as EntrezGene, RefSeq, GI, PDB, GO, PIR, NCBI-taxon, UniGene etc. each recorded as an identifier of the respective database. We also downloaded the Gene Ontology Protein Information Resource slim generic categories from the online GO database (Geneontology.org, (2014). *GO Database*. [online] Available at: http://www.geneontology.org/ontology/subsets/goslim_generic.obo [Accessed 19 June 2014].).

Using the mapping, GO slim and UniProt files together with the list of LIRCPs for a model organism, we generated GO class distribution files with counts of proteins having a particular GO slim category. One distribution file for each GO top level hierarchy (i.e., Biological Process, Cellular Component and Molecular Function) has been generated.

We assessed the statistical significance of each GO slim category of the model organisms using a hypergeometric test, employed through the Perl module Math::Pari (Search.cpan.org, (2014). Math-Pari-2.010808 Retrieved from: http://search.cpan.org/CPAN/authors/id/I/IL/ILYAZ/modules/Math-Pari-2.010808.zip.) based on the following criteria:

- Number of proteins assigned to a particular GO slim category in the model organism (n)
- Number of putative LIRCPs assigned to the same GO slim category in the model organism (x)
- Total number of proteins in the model organism (N)
- Total number of putative LIRCPs in the model organism (k)

The formula used for predicting the probability using hypergeometric test (h) is given below:^81^

$$\text{h}\left(x;N,n,k\right)\;=\;\left[{}_{\text{k}}{\text{C}}_{\text{x}}\right]\left[{}_{\text{N−k}}{\text{C}}_{\text{n−x}}\right]\text{/}[{}_{\text{N}}{\text{C}}_{\text{n}}].$$

To control the false discovery rate, we have also generated p-adjusted values employing the Benjamini-Hochberg method from Perl's Statistics::Multtest module (Search.cpan.org, (2014). Statistics-Multtest-0.13. Retrieved from: http://search.cpan.org/CPAN/authors/id/J/JO/JOKERGOO/Statistics-Multtest-0.13.tar.gz.). Following the hypergeometric test and false discovery rate correction, the GO distribution files were updated with p-value and p-adjusted values. Then, the GO slim categories data of model organisms was further classified based on different cut-offs for p-adjusted (p-adj) values as:

- Highly significant, (p-adj <= 0.01)
- Significant (p-adj > 0.01 and P-adj <= 0.05)
- Low significance (p-adj > 0.05 and p-adj <= 0.1)
- Not significant (p-adj > 0.1)

### Web application

The iLIR database has been developed for making the list of putative LIRCPs from the complete proteome of selected model organisms available for researchers worldwide. This web resource is based on well-established web technologies, including HTML, CSS, JavaScript, PHP (v5.3.28), JpGraph (v3.5.0b1) (Jpgraph.net, (2014). v3.5.0b1 Retrieved from: http://jpgraph.net/download/download.php?p=5) and the Apache web server technologies to develop and serve the web application.

## Supplementary Material

KAUP_A_1207016_Supplementary_material.zip

## Abbreviations

AIM: Atg8-interacting motif
GO: gene ontology
LIR: LC3-interacting region
LIRCP: LIR-containing protein
LRS: LC3 recognition sequence
MAP1LC3/LC3: microtubule associated protein 1 light chain 3
xLIR: extended LIR motif
