# Supplementary material for: iLIR database: A web resource for LIR motif-containing proteins in eukaryotes
Source: Autophagy. 2016 Aug 2;12(10):1945–53. doi: 10.1080/15548627.2016.1207016 (PMC5079668; doi:10.1080/15548627.2016.1207016)
Supplement: KAUP_A_1207016_Supplementary_material.zip [file kaup-12-10-1207016-s001.zip › KAUP_A_1207016 Supplementary material.pdf]

WARWICK
THE UNIVERSITY OF WARWICK

8 June 2016

Prof. Daniel Klionsky
Editor in Chief
Autophagy

Dear Dan,

We submit a revised version of our manuscript entitled ‘**iLIR database: a web resource for LIR motif-containing proteins in eukaryotes**’ by Jacomin et al.

In this version we have corrected the manuscript according to your comments and all names in the supplementary tables (symbols and full names) to match those in HGNC/MGI.

We hope that our work will meet all the criteria to be published in *Autophagy*.

Best regards,

Yannis

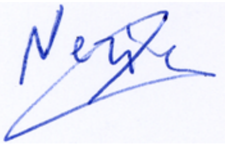

Dr Ioannis P. Nezis
Associate Professor
School of Life Sciences
University of Warwick
Gibbet Hill Campus
Coventry CV4 7AL, UK
Phone: +44 (0) 24 76 150400
E-mail: I.Nezis@warwick.ac.uk
http://www2.warwick.ac.uk/fac/sci/lifesci/people/inezis/
http://www2.warwick.ac.uk/fac/sci/lifesci/research/autophagy
http://autophagy.uk/

School of Life Sciences
The University of Warwick
Coventry CV4 7AL UK
T (0)24 76150400
F (0)24 7652 2052
E I.Nezis@warwick.ac.uk
www.warwick.ac.uk
