# Supplementary material for: iLIR database: A web resource for LIR motif-containing proteins in eukaryotes
Source: Autophagy. 2016 Aug 2;12(10):1945–53. doi: 10.1080/15548627.2016.1207016 (PMC5079668; doi:10.1080/15548627.2016.1207016)
Supplement: KAUP_A_1207016_Supplementary_material.zip [file kaup-12-10-1207016-s001.zip › 2016AUTO0134R2-s02.docx]

**SUPPLEMENTARY INFORMATION AND REFERENCES**

**iLIR database: a web resource for LIR motif-containing proteins in eukaryotes**

Anne-Claire Jacomin^1,¶^ , Siva Samavedam^1,¶^ , Vasilis Promponas^2,٭^ and Ioannis P. Nezis^1, ٭^

^1^School of Life Sciences, University of Warwick, CV4 7AL, Coventry, United Kingdom

^2^Bioinformatics Research Laboratory, Department of Biological Sciences, University of Cyprus, Nicosia, 1678, Cyprus

^¶^ equally first authors

٭ corresponding authors

**SUPPLEMENTARY FIGURE**

**Figure S1.** ‘GO Annotation’ result pages of the data available for the putative LIRCPs identified in the human proteome. This menu provides pre-computed information relative to the distribution of the GO term categories sorted for the putative LIRCPs identified for each model organism. (A) Screenshot of the GO Slim submenu result page. (B) Screenshot of the GO Slim classes’ distribution of the putative LIRCPs in the Molecular Function category.

**SUPPLEMENTARY TABLE LEGENDS**

**Suppl. Table 1.** List of the entries corresponding to the human proteins whose LIR motifs have been experimentally verified (group ‘A’). The table details the GO term categories for each protein for the three classes Molecular Function, Biological Process and Cellular Component. Each entry is identified by the gene symbol and name according to the organism nomenclature (HGNC and SGD for human and yeast entries respectively) that matches the corresponding Uniprot Accession number.

**Suppl. Table 2.** List of the proteins that have been shown to interact or colocalise with ATG8-family proteins (group ‘B’). The table shows the xLIR motifs (sequence, start position and PSSM score) for each protein, the ATG8-family member interaction partner, methods used to characterise the interaction and the role of the protein in autophagy. The proteins are sorted according to their main function, *i.e.* the selective autophagy receptors, the proteins implicated in the initiation of autophagy and formation of the autophagosomes, the proteins involved in the vesicular transport, the direct substrates for autophagic degradation. The ‘other’ category shows the proteins that do not enter any of the previous categories or whose function in autophagy has not yet be described. Each entry is identified by the gene symbol and name according to the organism nomenclature (HGNC and MGI for human and mouse entries respectively) that matches the corresponding Uniprot Accession number.

**Suppl. Table 3.** List of proteins that have been linked to autophagy or associated pathways (group ‘C’). The table shows 256 entries sorted for human and mouse proteins sharing their GO terms with the 21 experimentally verified LIRCPs (see Suppl. Table 1 and Figure 4). This table also includes the entries corresponding to the proteins listed in the Suppl. Table 2. Each entry is identified by the gene symbol and name according to the organism nomenclature (HGNC and MGI for human and mouse entries respectively) that matches the corresponding Uniprot Accession number.

**Suppl. Table 4.** List of the 756 entries from human proteome showing proteins that share the same GO terms as the experimentally verified LIRCPs but they have not been related to autophagy or associated pathways (group ‘D’). Each entry is identified by the gene symbol and name according to the HGNC nomenclature.

**SUPPLEMENTARY REFERENCES**

These references are related to the Supplementary Table 3.

Abreu, M. M. and L. Sealy (2010). "The C/EBPbeta isoform, liver-inhibitory protein (LIP), induces autophagy in breast cancer cell lines." Exp Cell Res **316**(19): 3227-3238.

Amarnath, S., F. A. Flomerfelt, C. M. Costanzo, J. E. Foley, J. Mariotti, D. M. Konecki, A. Gangopadhyay, M. Eckhaus, S. Wong, B. L. Levine, C. H. June and D. H. Fowler (2010). "Rapamycin generates anti-apoptotic human Th1/Tc1 cells via autophagy for induction of xenogeneic GVHD." Autophagy **6**(4): 523-541.

Artal-Martinez de Narvajas, A., T. S. Gomez, J. S. Zhang, A. O. Mann, Y. Taoda, J. A. Gorman, M. Herreros-Villanueva, T. M. Gress, V. Ellenrieder, L. Bujanda, D. H. Kim, A. P. Kozikowski, A. Koenig and D. D. Billadeau (2013). "Epigenetic regulation of autophagy by the methyltransferase G9a." Mol Cell Biol **33**(20): 3983-3993.

Bains, M., V. Zaegel, J. Mize-Berge and K. A. Heidenreich (2011). "IGF-I stimulates Rab7-RILP interaction during neuronal autophagy." Neurosci Lett **488**(2): 112-117.

Balakumaran, B. S., A. Porrello, D. S. Hsu, W. Glover, A. Foye, J. Y. Leung, B. A. Sullivan, W. C. Hahn, M. Loda and P. G. Febbo (2009). "MYC activity mitigates response to rapamycin in prostate cancer through eukaryotic initiation factor 4E-binding protein 1-mediated inhibition of autophagy." Cancer Res **69**(19): 7803-7810.

Beilina, A., I. N. Rudenko, A. Kaganovich, L. Civiero, H. Chau, S. K. Kalia, L. V. Kalia, E. Lobbestael, R. Chia, K. Ndukwe, J. Ding, M. A. Nalls, C. International Parkinson's Disease Genomics, C. North American Brain Expression, M. Olszewski, D. N. Hauser, R. Kumaran, A. M. Lozano, V. Baekelandt, L. E. Greene, J. M. Taymans, E. Greggio and M. R. Cookson (2014). "Unbiased screen for interactors of leucine-rich repeat kinase 2 supports a common pathway for sporadic and familial Parkinson disease." Proc Natl Acad Sci U S A **111**(7): 2626-2631.

Berezniuk, I., J. Sironi, M. B. Callaway, L. M. Castro, I. Y. Hirata, E. S. Ferro and L. D. Fricker (2010). "CCP1/Nna1 functions in protein turnover in mouse brain: Implications for cell death in Purkinje cell degeneration mice." FASEB J **24**(6): 1813-1823.

Biasoli, D., S. A. Kahn, T. A. Cornelio, M. Furtado, L. Campanati, H. Chneiweiss, V. Moura-Neto and H. L. Borges (2013). "Retinoblastoma protein regulates the crosstalk between autophagy and apoptosis, and favors glioblastoma resistance to etoposide." Cell Death Dis **4**: e767.

Bigford, G. E., O. F. Alonso, D. Dietrich and R. W. Keane (2009). "A novel protein complex in membrane rafts linking the NR2B glutamate receptor and autophagy is disrupted following traumatic brain injury." J Neurotrauma **26**(5): 703-720.

Boletta, A. (2009). "Emerging evidence of a link between the polycystins and the mTOR pathways." Pathogenetics **2**(1): 6.

Boon, J. Y., J. Dusonchet, C. Trengrove and B. Wolozin (2014). "Interaction of LRRK2 with kinase and GTPase signaling cascades." Front Mol Neurosci **7**: 64.

Bourke, L. T., R. A. Knight, D. S. Latchman, A. Stephanou and J. McCormick (2013). "Signal transducer and activator of transcription-1 localizes to the mitochondria and modulates mitophagy." JAKSTAT **2**(4): e25666.

Buraschi, S., T. Neill, A. Goyal, C. Poluzzi, J. Smythies, R. T. Owens, L. Schaefer, A. Torres and R. V. Iozzo (2013). "Decorin causes autophagy in endothelial cells via Peg3." Proc Natl Acad Sci U S A **110**(28): E2582-2591.

Cai, T., H. Hirai, G. Zhang, M. Zhang, N. Takahashi, H. Kasai, L. S. Satin, R. D. Leapman and A. L. Notkins (2011). "Deletion of Ia-2 and/or Ia-2beta in mice decreases insulin secretion by reducing the number of dense core vesicles." Diabetologia **54**(9): 2347-2357.

Carpenter, J. E. and C. Grose (2014). "Varicella-zoster virus glycoprotein expression differentially induces the unfolded protein response in infected cells." Front Microbiol **5**: 322.

Catalina-Rodriguez, O., V. K. Kolukula, Y. Tomita, A. Preet, F. Palmieri, A. Wellstein, S. Byers, A. J. Giaccia, E. Glasgow, C. Albanese and M. L. Avantaggiati (2012). "The mitochondrial citrate transporter, CIC, is essential for mitochondrial homeostasis." Oncotarget **3**(10): 1220-1235.

Cebotaru, V., L. Cebotaru, H. Kim, M. Chiaravalli, A. Boletta, F. Qian and W. B. Guggino (2014). "Polycystin-1 negatively regulates Polycystin-2 expression via the aggresome/autophagosome pathway." J Biol Chem **289**(10): 6404-6414.

Cenni, V., C. Capanni, M. Columbaro, M. Ortolani, M. R. D'Apice, G. Novelli, M. Fini, S. Marmiroli, E. Scarano, N. M. Maraldi, S. Squarzoni, S. Prencipe and G. Lattanzi (2011). "Autophagic degradation of farnesylated prelamin A as a therapeutic approach to lamin-linked progeria." Eur J Histochem **55**(4): e36.

Chang, C. P., Y. C. Su, C. W. Hu and H. Y. Lei (2013). "TLR2-dependent selective autophagy regulates NF-kappaB lysosomal degradation in hepatoma-derived M2 macrophage differentiation." Cell Death Differ **20**(3): 515-523.

Chang, S. H., S. H. Hong, H. L. Jiang, A. Minai-Tehrani, K. N. Yu, J. H. Lee, J. E. Kim, J. Y. Shin, B. Kang, S. Park, K. Han, C. Chae and M. H. Cho (2012). "GOLGA2/GM130, cis-Golgi matrix protein, is a novel target of anticancer gene therapy." Mol Ther **20**(11): 2052-2063.

Chang, Y. P., C. C. Tsai, W. C. Huang, C. Y. Wang, C. L. Chen, Y. S. Lin, J. I. Kai, C. Y. Hsieh, Y. L. Cheng, P. C. Choi, S. H. Chen, S. P. Chang, H. S. Liu and C. F. Lin (2010). "Autophagy facilitates IFN-gamma-induced Jak2-STAT1 activation and cellular inflammation." J Biol Chem **285**(37): 28715-28722.

Chikh, A., P. Sanza, C. Raimondi, O. Akinduro, G. Warnes, G. Chiorino, C. Byrne, C. A. Harwood and D. Bergamaschi (2014). "iASPP is a novel autophagy inhibitor in keratinocytes." J Cell Sci **127**(Pt 14): 3079-3093.

Cho, S. J., S. M. Yun, C. Jo, D. H. Lee, K. J. Choi, J. C. Song, S. I. Park, Y. J. Kim and Y. H. Koh (2015). "SUMO1 promotes Abeta production via the modulation of autophagy." Autophagy **11**(1): 100-112.

Chong-Kopera, H., K. Inoki, Y. Li, T. Zhu, F. R. Garcia-Gonzalo, J. L. Rosa and K. L. Guan (2006). "TSC1 stabilizes TSC2 by inhibiting the interaction between TSC2 and the HERC1 ubiquitin ligase." J Biol Chem **281**(13): 8313-8316.

Colleran, A., A. Ryan, A. O'Gorman, C. Mureau, C. Liptrot, P. Dockery, H. Fearnhead and L. J. Egan (2011). "Autophagosomal IkappaB alpha degradation plays a role in the long term control of tumor necrosis factor-alpha-induced nuclear factor-kappaB (NF-kappaB) activity." J Biol Chem **286**(26): 22886-22893.

Couve, A., S. Restituito, J. M. Brandon, K. J. Charles, H. Bawagan, K. B. Freeman, M. N. Pangalos, A. R. Calver and S. J. Moss (2004). "Marlin-1, a novel RNA-binding protein associates with GABA receptors." J Biol Chem **279**(14): 13934-13943.

Cullinane, A. R., A. A. Schaffer and M. Huizing (2013). "The BEACH is hot: a LYST of emerging roles for BEACH-domain containing proteins in human disease." Traffic **14**(7): 749-766.

Dunlop, E. A., S. Seifan, T. Claessens, C. Behrends, M. A. Kamps, E. Rozycka, A. J. Kemp, R. K. Nookala, J. Blenis, B. J. Coull, J. T. Murray, M. A. van Steensel, S. Wilkinson and A. R. Tee (2014). "FLCN, a novel autophagy component, interacts with GABARAP and is regulated by ULK1 phosphorylation." Autophagy **10**(10): 1749-1760.

Esselens, C., V. Oorschot, V. Baert, T. Raemaekers, K. Spittaels, L. Serneels, H. Zheng, P. Saftig, B. De Strooper, J. Klumperman and W. Annaert (2004). "Presenilin 1 mediates the turnover of telencephalin in hippocampal neurons via an autophagic degradative pathway." J Cell Biol **166**(7): 1041-1054.

Esteve, J. M., M. E. Armengod and E. Knecht (2010). "BRCA1 negatively regulates formation of autophagic vacuoles in MCF-7 breast cancer cells." Exp Cell Res **316**(16): 2618-2629.

Fan, S., Q. Meng, T. Saha, F. H. Sarkar and E. M. Rosen (2009). "Low concentrations of diindolylmethane, a metabolite of indole-3-carbinol, protect against oxidative stress in a BRCA1-dependent manner." Cancer Res **69**(15): 6083-6091.

Fremont, S., A. Gerard, M. Galloux, K. Janvier, R. E. Karess and C. Berlioz-Torrent (2013). "Beclin-1 is required for chromosome congression and proper outer kinetochore assembly." EMBO Rep **14**(4): 364-372.

Fu, M. M., J. J. Nirschl and E. L. Holzbaur (2014). "LC3 binding to the scaffolding protein JIP1 regulates processive dynein-driven transport of autophagosomes." Dev Cell **29**(5): 577-590.

Fung, C., X. Chen, J. R. Grandis and U. Duvvuri (2012). "EGFR tyrosine kinase inhibition induces autophagy in cancer cells." Cancer Biol Ther **13**(14): 1417-1424.

Gamerdinger, M., A. M. Kaya, U. Wolfrum, A. M. Clement and C. Behl (2011). "BAG3 mediates chaperone-based aggresome-targeting and selective autophagy of misfolded proteins." EMBO Rep **12**(2): 149-156.

Garcia-Navas, R., M. Munder and F. Mollinedo (2012). "Depletion of L-arginine induces autophagy as a cytoprotective response to endoplasmic reticulum stress in human T lymphocytes." Autophagy **8**(11): 1557-1576.

Ghavami, S., M. Eshragi, S. R. Ande, W. J. Chazin, T. Klonisch, A. J. Halayko, K. D. McNeill, M. Hashemi, C. Kerkhoff and M. Los (2010). "S100A8/A9 induces autophagy and apoptosis via ROS-mediated cross-talk between mitochondria and lysosomes that involves BNIP3." Cell Res **20**(3): 314-331.

Gibbings, D., S. Mostowy, F. Jay, Y. Schwab, P. Cossart and O. Voinnet (2012). "Selective autophagy degrades DICER and AGO2 and regulates miRNA activity." Nat Cell Biol **14**(12): 1314-1321.

Goyal, A., T. Neill, R. T. Owens, L. Schaefer and R. V. Iozzo (2014). "Decorin activates AMPK, an energy sensor kinase, to induce autophagy in endothelial cells." Matrix Biol **34**: 46-54.

Green, A. M., P. R. Beatty, A. Hadjilaou and E. Harris (2014). "Innate immunity to dengue virus infection and subversion of antiviral responses." J Mol Biol **426**(6): 1148-1160.

Guo, L., J. X. Huang, Y. Liu, X. Li, S. R. Zhou, S. W. Qian, Y. Liu, H. Zhu, H. Y. Huang, Y. J. Dang and Q. Q. Tang (2013). "Transactivation of Atg4b by C/EBPbeta promotes autophagy to facilitate adipogenesis." Mol Cell Biol **33**(16): 3180-3190.

Heldring, N., U. Nyman, P. Lonnerberg, S. Onnestam, A. Herland, J. Holmberg and O. Hermanson (2014). "NCoR controls glioblastoma tumor cell characteristics." Neuro Oncol **16**(2): 241-249.

Hong, J. H., L. Kaustov, E. Coyaud, T. Srikumar, J. Wan, C. Arrowsmith and B. Raught (2015). "KCMF1 links RAD6 to UBR4 and lysosome-mediated degradation." Mol Cell Proteomics.

Hoyer-Hansen, M., L. Bastholm, P. Szyniarowski, M. Campanella, G. Szabadkai, T. Farkas, K. Bianchi, N. Fehrenbacher, F. Elling, R. Rizzuto, I. S. Mathiasen and M. Jaattela (2007). "Control of macroautophagy by calcium, calmodulin-dependent kinase kinase-beta, and Bcl-2." Mol Cell **25**(2): 193-205.

Huang, C., M. Z. Lin, D. Cheng, F. Braet, C. A. Pollock and X. M. Chen (2014). "Thioredoxin-interacting protein mediates dysfunction of tubular autophagy in diabetic kidneys through inhibiting autophagic flux." Lab Invest **94**(3): 309-320.

Huang, X., Z. Wu, Y. Mei and M. Wu (2013). "XIAP inhibits autophagy via XIAP-Mdm2-p53 signalling." EMBO J **32**(16): 2204-2216.

Huttenhower, C., E. M. Haley, M. A. Hibbs, V. Dumeaux, D. R. Barrett, H. A. Coller and O. G. Troyanskaya (2009). "Exploring the human genome with functional maps." Genome Res **19**(6): 1093-1106.

Infante, A., A. Gago, G. R. de Eguino, T. Calvo-Fernandez, V. Gomez-Vallejo, J. Llop, K. Schlangen, A. Fullaondo, A. M. Aransay, A. Martin and C. I. Rodriguez (2014). "Prelamin A accumulation and stress conditions induce impaired Oct-1 activity and autophagy in prematurely aged human mesenchymal stem cell." Aging (Albany NY) **6**(4): 264-280.

Isakson, P., M. Bjoras, S. O. Boe and A. Simonsen (2010). "Autophagy contributes to therapy-induced degradation of the PML/RARA oncoprotein." Blood **116**(13): 2324-2331.

Isakson, P., A. H. Lystad, K. Breen, G. Koster, H. Stenmark and A. Simonsen (2013). "TRAF6 mediates ubiquitination of KIF23/MKLP1 and is required for midbody ring degradation by selective autophagy." Autophagy **9**(12): 1955-1964.

Jiang, H., V. Martin, C. Gomez-Manzano, D. G. Johnson, M. Alonso, E. White, J. Xu, T. J. McDonnell, N. Shinojima and J. Fueyo (2010). "The RB-E2F1 pathway regulates autophagy." Cancer Res **70**(20): 7882-7893.

Jimenez-Sanchez, M., F. M. Menzies, Y. Y. Chang, N. Simecek, T. P. Neufeld and D. C. Rubinsztein (2012). "The Hedgehog signalling pathway regulates autophagy." Nat Commun **3**: 1200.

Jones, S., D. L. Cunningham, J. Z. Rappoport and J. K. Heath (2014). "The non-receptor tyrosine kinase Ack1 regulates the fate of activated EGFR by inducing trafficking to the p62/NBR1 pre-autophagosome." J Cell Sci **127**(Pt 5): 994-1006.

Jungbluth, H. and M. Gautel (2014). "Pathogenic mechanisms in centronuclear myopathies." Front Aging Neurosci **6**: 339.

Kang, Z. H., C. Y. Wang, W. L. Zhang, J. T. Zhang, C. H. Yuan, P. W. Zhao, Y. Y. Lin, S. Hong, C. Y. Li and L. Wang (2014). "Histone deacetylase HDAC4 promotes gastric cancer SGC-7901 cells progression via p21 repression." PLoS One **9**(6): e98894.

Kenzelmann Broz, D. and L. D. Attardi (2013). "TRP53 activates a global autophagy program to promote tumor suppression." Autophagy **9**(9): 1440-1442.

Kim, T. S., M. Kawaguchi, M. Suzuki, C. G. Jung, K. Asai, Y. Shibamoto, M. F. Lavin, K. K. Khanna and Y. Miura (2010). "The ZFHX3 (ATBF1) transcription factor induces PDGFRB, which activates ATM in the cytoplasm to protect cerebellar neurons from oxidative stress." Dis Model Mech **3**(11-12): 752-762.

Kim, Y., Y. S. Kim, D. E. Kim, J. S. Lee, J. H. Song, H. G. Kim, D. H. Cho, S. Y. Jeong, D. H. Jin, S. J. Jang, H. S. Seol, Y. A. Suh, S. J. Lee, C. S. Kim, J. Y. Koh and J. J. Hwang (2013). "BIX-01294 induces autophagy-associated cell death via EHMT2/G9a dysfunction and intracellular reactive oxygen species production." Autophagy **9**(12): 2126-2139.

Knaevelsrud, H., K. Soreng, C. Raiborg, K. Haberg, F. Rasmuson, A. Brech, K. Liestol, T. E. Rusten, H. Stenmark, T. P. Neufeld, S. R. Carlsson and A. Simonsen (2013). "Membrane remodeling by the PX-BAR protein SNX18 promotes autophagosome formation." J Cell Biol **202**(2): 331-349.

Kobuna, H., T. Inoue, M. Shibata, K. Gengyo-Ando, A. Yamamoto, S. Mitani and H. Arai (2010). "Multivesicular body formation requires OSBP-related proteins and cholesterol." PLoS Genet **6**(8).

Kouroku, Y., E. Fujita, I. Tanida, T. Ueno, A. Isoai, H. Kumagai, S. Ogawa, R. J. Kaufman, E. Kominami and T. Momoi (2007). "ER stress (PERK/eIF2alpha phosphorylation) mediates the polyglutamine-induced LC3 conversion, an essential step for autophagy formation." Cell Death Differ **14**(2): 230-239.

Kuchay, S., S. Duan, E. Schenkein, A. Peschiaroli, A. Saraf, L. Florens, M. P. Washburn and M. Pagano (2013). "FBXL2- and PTPL1-mediated degradation of p110-free p85beta regulatory subunit controls the PI(3)K signalling cascade." Nat Cell Biol **15**(5): 472-480.

Laane, E., K. P. Tamm, E. Buentke, K. Ito, P. Kharaziha, J. Oscarsson, M. Corcoran, A. C. Bjorklund, K. Hultenby, J. Lundin, M. Heyman, S. Soderhall, J. Mazur, A. Porwit, P. P. Pandolfi, B. Zhivotovsky, T. Panaretakis and D. Grander (2009). "Cell death induced by dexamethasone in lymphoid leukemia is mediated through initiation of autophagy." Cell Death Differ **16**(7): 1018-1029.

Laurila, E., E. Vuorinen, K. Savinainen, H. Rauhala and A. Kallioniemi (2014). "KPNA7, a nuclear transport receptor, promotes malignant properties of pancreatic cancer cells in vitro." Exp Cell Res **322**(1): 159-167.

Li, G., J. Peng, Y. Liu, X. Li, Q. Yang, Y. Li, Z. Tang, Z. Wang, Z. Jiang and D. Wei (2015). "Oxidized low-density lipoprotein inhibits THP-1-derived macrophage autophagy via TET2 down-regulation." Lipids **50**(2): 177-183.

Li, H., S. Huang, S. Wang, L. Wang, L. Qi, Y. Zhang, S. Zhang, B. Zhao and J. Miao (2013). "Relationship between annexin A7 and integrin beta4 in autophagy." Int J Biochem Cell Biol **45**(11): 2605-2611.

Li, L. and K. L. Guan (2013). "Microtubule-associated protein/microtubule affinity-regulating kinase 4 (MARK4) is a negative regulator of the mammalian target of rapamycin complex 1 (mTORC1)." J Biol Chem **288**(1): 703-708.

Li, P., Q. Du, Z. Cao, Z. Guo, J. Evankovich, W. Yan, Y. Chang, L. Shao, D. B. Stolz, A. Tsung and D. A. Geller (2012). "Interferon-gamma induces autophagy with growth inhibition and cell death in human hepatocellular carcinoma (HCC) cells through interferon-regulatory factor-1 (IRF-1)." Cancer Lett **314**(2): 213-222.

Lin, F., G. Ghislat, S. Luo, M. Renna, F. Siddiqi and D. C. Rubinsztein (2015). "XIAP and cIAP1 amplifications induce Beclin 1-dependent autophagy through NFkappaB activation." Hum Mol Genet.

Liu, H., P. Wang, W. Song and X. Sun (2009). "Degradation of regulator of calcineurin 1 (RCAN1) is mediated by both chaperone-mediated autophagy and ubiquitin proteasome pathways." FASEB J **23**(10): 3383-3392.

Liu, L., W. L. McKeehan, F. Wang and R. Xie (2012). "MAP1S enhances autophagy to suppress tumorigenesis." Autophagy **8**(2): 278-280.

Lo Re, A. E., M. G. Fernandez-Barrena, L. L. Almada, L. D. Mills, S. F. Elsawa, G. Lund, A. Ropolo, M. I. Molejon, M. I. Vaccaro and M. E. Fernandez-Zapico (2012). "Novel AKT1-GLI3-VMP1 pathway mediates KRAS oncogene-induced autophagy in cancer cells." J Biol Chem **287**(30): 25325-25334.

Longatti, A., C. A. Lamb, M. Razi, S. Yoshimura, F. A. Barr and S. A. Tooze (2012). "TBC1D14 regulates autophagosome formation via Rab11- and ULK1-positive recycling endosomes." J Cell Biol **197**(5): 659-675.

Lopez-Herrera, G., G. Tampella, Q. Pan-Hammarstrom, P. Herholz, C. M. Trujillo-Vargas, K. Phadwal, A. K. Simon, M. Moutschen, A. Etzioni, A. Mory, I. Srugo, D. Melamed, K. Hultenby, C. Liu, M. Baronio, M. Vitali, P. Philippet, V. Dideberg, A. Aghamohammadi, N. Rezaei, V. Enright, L. Du, U. Salzer, H. Eibel, D. Pfeifer, H. Veelken, H. Stauss, V. Lougaris, A. Plebani, E. M. Gertz, A. A. Schaffer, L. Hammarstrom and B. Grimbacher (2012). "Deleterious mutations in LRBA are associated with a syndrome of immune deficiency and autoimmunity." Am J Hum Genet **90**(6): 986-1001.

Ma, D., S. Panda and J. D. Lin (2011). "Temporal orchestration of circadian autophagy rhythm by C/EBPbeta." EMBO J **30**(22): 4642-4651.

Ma, S. and B. P. Rubin (2014). "Apoptosis-associated tyrosine kinase 1 inhibits growth and migration and promotes apoptosis in melanoma." Lab Invest **94**(4): 430-438.

Mashimo, T., O. Hadjebi, F. Amair-Pinedo, T. Tsurumi, F. Langa, T. Serikawa, C. Sotelo, J. L. Guenet and J. L. Rosa (2009). "Progressive Purkinje cell degeneration in tambaleante mutant mice is a consequence of a missense mutation in HERC1 E3 ubiquitin ligase." PLoS Genet **5**(12): e1000784.

Matsunaga, K., T. Saitoh, K. Tabata, H. Omori, T. Satoh, N. Kurotori, I. Maejima, K. Shirahama-Noda, T. Ichimura, T. Isobe, S. Akira, T. Noda and T. Yoshimori (2009). "Two Beclin 1-binding proteins, Atg14L and Rubicon, reciprocally regulate autophagy at different stages." Nat Cell Biol **11**(4): 385-396.

Metge, B. J., A. Mitra, D. Chen, L. A. Shevde and R. S. Samant (2015). "N-Myc and STAT Interactor regulates autophagy and chemosensitivity in breast cancer cells." Sci Rep **5**: 11995.

Molitoris, J. K., K. S. McColl, S. Swerdlow, M. Matsuyama, M. Lam, T. H. Finkel, S. Matsuyama and C. W. Distelhorst (2011). "Glucocorticoid elevation of dexamethasone-induced gene 2 (Dig2/RTP801/REDD1) protein mediates autophagy in lymphocytes." J Biol Chem **286**(34): 30181-30189.

Moriyama, M., H. Moriyama, J. Uda, A. Matsuyama, M. Osawa and T. Hayakawa (2014). "BNIP3 plays crucial roles in the differentiation and maintenance of epidermal keratinocytes." J Invest Dermatol **134**(6): 1627-1635.

Mousavi, S. A., R. Kjeken, T. O. Berg, P. O. Seglen, T. Berg and A. Brech (2001). "Effects of inhibitors of the vacuolar proton pump on hepatic heterophagy and autophagy." Biochim Biophys Acta **1510**(1-2): 243-257.

N'Diaye, E. N., K. K. Kajihara, I. Hsieh, H. Morisaki, J. Debnath and E. J. Brown (2009). "PLIC proteins or ubiquilins regulate autophagy-dependent cell survival during nutrient starvation." EMBO Rep **10**(2): 173-179.

Naydenov, N. G., G. Harris, V. Morales and A. I. Ivanov (2012). "Loss of a membrane trafficking protein alphaSNAP induces non-canonical autophagy in human epithelia." Cell Cycle **11**(24): 4613-4625.

Nick, A. M., R. L. Stone, G. Armaiz-Pena, B. Ozpolat, I. Tekedereli, W. S. Graybill, C. N. Landen, G. Villares, P. Vivas-Mejia, J. Bottsford-Miller, H. S. Kim, J. S. Lee, S. M. Kim, K. A. Baggerly, P. T. Ram, M. T. Deavers, R. L. Coleman, G. Lopez-Berestein and A. K. Sood (2011). "Silencing of p130cas in ovarian carcinoma: a novel mechanism for tumor cell death." J Natl Cancer Inst **103**(21): 1596-1612.

Niso-Santano, M., S. Shen, S. Adjemian, S. A. Malik, G. Marino, S. Lachkar, L. Senovilla, O. Kepp, L. Galluzzi, M. C. Maiuri and G. Kroemer (2013). "Direct interaction between STAT3 and EIF2AK2 controls fatty acid-induced autophagy." Autophagy **9**(3): 415-417.

Ochaba, J., T. Lukacsovich, G. Csikos, S. Zheng, J. Margulis, L. Salazar, K. Mao, A. L. Lau, S. Y. Yeung, S. Humbert, F. Saudou, D. J. Klionsky, S. Finkbeiner, S. O. Zeitlin, J. L. Marsh, D. E. Housman, L. M. Thompson and J. S. Steffan (2014). "Potential function for the Huntingtin protein as a scaffold for selective autophagy." Proc Natl Acad Sci U S A.

Ohta, K., A. Mizuno, M. Ueda, S. Li, Y. Suzuki, Y. Hida, Y. Hayakawa-Yano, M. Itoh, E. Ohta, M. Kobori and T. Nakagawa (2010). "Autophagy impairment stimulates PS1 expression and gamma-secretase activity." Autophagy **6**(3): 345-352.

Pacheco, C. D., M. J. Elrick and A. P. Lieberman (2009). "Tau deletion exacerbates the phenotype of Niemann-Pick type C mice and implicates autophagy in pathogenesis." Hum Mol Genet **18**(5): 956-965.

Palomer, X., E. Capdevila-Busquets, G. Botteri, L. Salvado, E. Barroso, M. M. Davidson, L. Michalik, W. Wahli and M. Vazquez-Carrera (2014). "PPARbeta/delta attenuates palmitate-induced endoplasmic reticulum stress and induces autophagic markers in human cardiac cells." Int J Cardiol **174**(1): 110-118.

Parkhitko, A., F. Myachina, T. A. Morrison, K. M. Hindi, N. Auricchio, M. Karbowniczek, J. J. Wu, T. Finkel, D. J. Kwiatkowski, J. J. Yu and E. P. Henske (2011). "Tumorigenesis in tuberous sclerosis complex is autophagy and p62/sequestosome 1 (SQSTM1)-dependent." Proc Natl Acad Sci U S A **108**(30): 12455-12460.

Paul, S., A. K. Kashyap, W. Jia, Y. W. He and B. C. Schaefer (2012). "Selective autophagy of the adaptor protein Bcl10 modulates T cell receptor activation of NF-kappaB." Immunity **36**(6): 947-958.

Polager, S., M. Ofir and D. Ginsberg (2008). "E2F1 regulates autophagy and the transcription of autophagy genes." Oncogene **27**(35): 4860-4864.

Qing, G., P. Yan, Z. Qu, H. Liu and G. Xiao (2007). "Hsp90 regulates processing of NF-kappa B2 p100 involving protection of NF-kappa B-inducing kinase (NIK) from autophagy-mediated degradation." Cell Res **17**(6): 520-530.

Ramirez-Valle, F., S. Braunstein, J. Zavadil, S. C. Formenti and R. J. Schneider (2008). "eIF4GI links nutrient sensing by mTOR to cell proliferation and inhibition of autophagy." J Cell Biol **181**(2): 293-307.

Rapino, F., M. Jung and S. Fulda (2014). "BAG3 induction is required to mitigate proteotoxicity via selective autophagy following inhibition of constitutive protein degradation pathways." Oncogene **33**(13): 1713-1724.

Renna, M., C. F. Bento, A. Fleming, F. M. Menzies, F. H. Siddiqi, B. Ravikumar, C. Puri, M. Garcia-Arencibia, O. Sadiq, S. Corrochano, S. Carter, S. D. Brown, A. Acevedo-Arozena and D. C. Rubinsztein (2013). "IGF-1 receptor antagonism inhibits autophagy." Hum Mol Genet **22**(22): 4528-4544.

Rose, J. M., S. S. Novoselov, P. A. Robinson and M. E. Cheetham (2011). "Molecular chaperone-mediated rescue of mitophagy by a Parkin RING1 domain mutant." Hum Mol Genet **20**(1): 16-27.

Ryu, H. J., J. E. Kim, S. I. Yeo and T. C. Kang (2011). "p65/RelA-Ser529 NF-kappaB subunit phosphorylation induces autophagic astroglial death (Clasmatodendrosis) following status epilepticus." Cell Mol Neurobiol **31**(7): 1071-1078.

Rzymski, T., M. Milani, D. C. Singleton and A. L. Harris (2009). "Role of ATF4 in regulation of autophagy and resistance to drugs and hypoxia." Cell Cycle **8**(23): 3838-3847.

Salewsky, B., P. Wessendorf, D. Hirsch, H. Krenzlin and M. Digweed (2013). "Nijmegen breakage syndrome: the clearance pathway for mutant nibrin protein is allele specific." Gene **519**(2): 217-221.

Sariyer, I. K., N. Merabova, P. K. Patel, T. Knezevic, A. Rosati, M. C. Turco and K. Khalili (2012). "Bag3-induced autophagy is associated with degradation of JCV oncoprotein, T-Ag." PLoS One **7**(9): e45000.

Sarkar, C., Z. Zhao, S. Aungst, B. Sabirzhanov, A. I. Faden and M. M. Lipinski (2014). "Impaired autophagy flux is associated with neuronal cell death after traumatic brain injury." Autophagy **10**(12): 2208-2222.

Schultz, M. L., L. Tecedor, C. S. Stein, M. A. Stamnes and B. L. Davidson (2014). "CLN3 deficient cells display defects in the ARF1-Cdc42 pathway and actin-dependent events." PLoS One **9**(5): e96647.

Schulze, R. J., S. G. Weller, B. Schroeder, E. W. Krueger, S. Chi, C. A. Casey and M. A. McNiven (2013). "Lipid droplet breakdown requires dynamin 2 for vesiculation of autolysosomal tubules in hepatocytes." J Cell Biol **203**(2): 315-326.

Sebti, S., C. Prebois, E. Perez-Gracia, C. Bauvy, F. Desmots, N. Pirot, C. Gongora, A. S. Bach, A. V. Hubberstey, V. Palissot, G. Berchem, P. Codogno, L. K. Linares, E. Liaudet-Coopman and S. Pattingre (2014). "BAG6/BAT3 modulates autophagy by affecting EP300/p300 intracellular localization." Autophagy **10**(7): 1341-1342.

Sebti, S., C. Prebois, E. Perez-Gracia, C. Bauvy, F. Desmots, N. Pirot, C. Gongora, A. S. Bach, A. V. Hubberstey, V. Palissot, G. Berchem, P. Codogno, L. K. Linares, E. Liaudet-Coopman and S. Pattingre (2014). "BAT3 modulates p300-dependent acetylation of p53 and autophagy-related protein 7 (ATG7) during autophagy." Proc Natl Acad Sci U S A **111**(11): 4115-4120.

Settembre, C., C. Di Malta, V. A. Polito, M. Garcia Arencibia, F. Vetrini, S. Erdin, S. U. Erdin, T. Huynh, D. Medina, P. Colella, M. Sardiello, D. C. Rubinsztein and A. Ballabio (2011). "TFEB links autophagy to lysosomal biogenesis." Science **332**(6036): 1429-1433.

Shen, S., M. Niso-Santano, S. Adjemian, T. Takehara, S. A. Malik, H. Minoux, S. Souquere, G. Marino, S. Lachkar, L. Senovilla, L. Galluzzi, O. Kepp, G. Pierron, M. C. Maiuri, H. Hikita, R. Kroemer and G. Kroemer (2012). "Cytoplasmic STAT3 represses autophagy by inhibiting PKR activity." Mol Cell **48**(5): 667-680.

Sun, Q., J. Zhang, W. Fan, K. N. Wong, X. Ding, S. Chen and Q. Zhong (2011). "The RUN domain of rubicon is important for hVps34 binding, lipid kinase inhibition, and autophagy suppression." J Biol Chem **286**(1): 185-191.

Sun, W., Y. Zheng, Z. Lu, Y. Cui, Q. Tian, S. Xiao, F. Liu and J. Liu (2014). "Overexpression of S100A7 protects LPS-induced mitochondrial dysfunction and stimulates IL-6 and IL-8 in HaCaT cells." PLoS One **9**(3): e92927.

Sun, Y., W. Guo, T. Ren, W. Liang, W. Zhou, Q. Lu, G. Jiao and T. Yan (2014). "Gli1 inhibition suppressed cell growth and cell cycle progression and induced apoptosis as well as autophagy depending on ERK1/2 activity in human chondrosarcoma cells." Cell Death Dis **5**: e979.

Tabata, K., K. Matsunaga, A. Sakane, T. Sasaki, T. Noda and T. Yoshimori (2010). "Rubicon and PLEKHM1 negatively regulate the endocytic/autophagic pathway via a novel Rab7-binding domain." Mol Biol Cell **21**(23): 4162-4172.

Talloczy, Z., W. Jiang, H. W. t. Virgin, D. A. Leib, D. Scheuner, R. J. Kaufman, E. L. Eskelinen and B. Levine (2002). "Regulation of starvation- and virus-induced autophagy by the eIF2alpha kinase signaling pathway." Proc Natl Acad Sci U S A **99**(1): 190-195.

Tang, Z., M. G. Lin, T. R. Stowe, S. Chen, M. Zhu, T. Stearns, B. Franco and Q. Zhong (2013). "Autophagy promotes primary ciliogenesis by removing OFD1 from centriolar satellites." Nature **502**(7470): 254-257.

Tsuneoka, M., T. Umata, H. Kimura, Y. Koda, M. Nakajima, K. Kosai, T. Takahashi, Y. Takahashi and A. Yamamoto (2003). "c-myc induces autophagy in rat 3Y1 fibroblast cells." Cell Struct Funct **28**(3): 195-204.

Tumbarello, D. A., B. J. Waxse, S. D. Arden, N. A. Bright, J. Kendrick-Jones and F. Buss (2012). "Autophagy receptors link myosin VI to autophagosomes to mediate Tom1-dependent autophagosome maturation and fusion with the lysosome." Nat Cell Biol **14**(10): 1024-1035.

Ulbricht, A., F. J. Eppler, V. E. Tapia, P. F. van der Ven, N. Hampe, N. Hersch, P. Vakeel, D. Stadel, A. Haas, P. Saftig, C. Behrends, D. O. Furst, R. Volkmer, B. Hoffmann, W. Kolanus and J. Hohfeld (2013). "Cellular mechanotransduction relies on tension-induced and chaperone-assisted autophagy." Curr Biol **23**(5): 430-435.

von Gunten, S. and H. U. Simon (2007). "Autophagic-like cell death in neutrophils induced by autoantibodies." Autophagy **3**(1): 67-68.

Walch, L. (2013). "Emerging role of the scaffolding protein Dlg1 in vesicle trafficking." Traffic **14**(9): 964-973.

Wang, B., S. Ling and W. C. Lin (2010). "14-3-3Tau regulates Beclin 1 and is required for autophagy." PLoS One **5**(4): e10409.

Wang, J., R. Kang, H. Huang, X. Xi, B. Wang, J. Wang and Z. Zhao (2014). "Hepatitis C virus core protein activates autophagy through EIF2AK3 and ATF6 UPR pathway-mediated MAP1LC3B and ATG12 expression." Autophagy **10**(5): 766-784.

Wang, J. W., J. Howson, E. Haller and W. G. Kerr (2001). "Identification of a novel lipopolysaccharide-inducible gene with key features of both A kinase anchor proteins and chs1/beige proteins." J Immunol **166**(7): 4586-4595.

Wang, Y., W. Wang, D. Li, M. Li, P. Wang, J. Wen, M. Liang, B. Su and Y. Yin (2014). "IGF-1 Alleviates NMDA-Induced Excitotoxicity in Cultured Hippocampal Neurons Against Autophagy via the NR2B/PI3K-AKT-mTOR Pathway." J Cell Physiol **229**(11): 1618-1629.

Webber, E., L. Li and L. S. Chin (2008). "Hypertonia-associated protein Trak1 is a novel regulator of endosome-to-lysosome trafficking." J Mol Biol **382**(3): 638-651.

Wei, Y., Z. Zou, N. Becker, M. Anderson, R. Sumpter, G. Xiao, L. Kinch, P. Koduru, C. S. Christudass, R. W. Veltri, N. V. Grishin, M. Peyton, J. Minna, G. Bhagat and B. Levine (2013). "EGFR-mediated Beclin 1 phosphorylation in autophagy suppression, tumor progression, and tumor chemoresistance." Cell **154**(6): 1269-1284.

Wen, Y., B. Zand, B. Ozpolat, M. J. Szczepanski, C. Lu, E. Yuca, A. R. Carroll, N. Alpay, C. Bartholomeusz, I. Tekedereli, Y. Kang, R. Rupaimoole, C. V. Pecot, H. J. Dalton, A. Hernandez, A. Lokshin, S. K. Lutgendorf, J. Liu, W. N. Hittelman, W. Y. Chen, G. Lopez-Berestein, M. Szajnik, N. T. Ueno, R. L. Coleman and A. K. Sood (2014). "Antagonism of tumoral prolactin receptor promotes autophagy-related cell death." Cell Rep **7**(2): 488-500.

Woldt, E., Y. Sebti, L. A. Solt, C. Duhem, S. Lancel, J. Eeckhoute, M. K. Hesselink, C. Paquet, S. Delhaye, Y. Shin, T. M. Kamenecka, G. Schaart, P. Lefebvre, R. Neviere, T. P. Burris, P. Schrauwen, B. Staels and H. Duez (2013). "Rev-erb-alpha modulates skeletal muscle oxidative capacity by regulating mitochondrial biogenesis and autophagy." Nat Med **19**(8): 1039-1046.

Yan, J., Z. Y. Wang, H. Z. Yang, H. Z. Liu, S. Mi, X. X. Lv, X. M. Fu, H. M. Yan, X. W. Zhang, Q. M. Zhan and Z. W. Hu (2011). "Timing is critical for an effective anti-metastatic immunotherapy: the decisive role of IFNgamma/STAT1-mediated activation of autophagy." PLoS One **6**(9): e24705.

Yang, F. C., B. C. Tan, W. H. Chen, Y. H. Lin, J. Y. Huang, H. Y. Chang, H. Y. Sun, P. H. Hsu, G. G. Liou, J. Shen, C. J. Chang, C. C. Han, M. D. Tsai and S. C. Lee (2013). "Reversible acetylation regulates salt-inducible kinase (SIK2) and its function in autophagy." J Biol Chem **288**(9): 6227-6237.

Yao, J., K. Zheng, C. Li, H. Liu and X. Shan (2015). "Interference of Notch1 inhibits the growth of glioma cancer cells by inducing cell autophagy and down-regulation of Notch1-Hes-1 signaling pathway." Med Oncol **32**(6): 610.

Yin, L., S. Kharbanda and D. Kufe (2009). "MUC1 oncoprotein promotes autophagy in a survival response to glucose deprivation." Int J Oncol **34**(6): 1691-1699.

Younce, C. and P. Kolattukudy (2012). "MCP-1 induced protein promotes adipogenesis via oxidative stress, endoplasmic reticulum stress and autophagy." Cell Physiol Biochem **30**(2): 307-320.

Yu, L., V. Tumati, S. F. Tseng, F. M. Hsu, D. N. Kim, D. Hong, J. T. Hsieh, C. Jacobs, P. Kapur and D. Saha (2012). "DAB2IP regulates autophagy in prostate cancer in response to combined treatment of radiation and a DNA-PKcs inhibitor." Neoplasia **14**(12): 1203-1212.

Zhang, G., J. Liu, Y. Zhang, J. Qu, L. Xu, H. Zheng, Y. Liu and X. Qu (2012). "Cbl-b-dependent degradation of FLIP(L) is involved in ATO-induced autophagy in leukemic K562 and gastric cancer cells." FEBS Lett **586**(19): 3104-3110.

Zhang, J., J. Kim, A. Alexander, S. Cai, D. N. Tripathi, R. Dere, A. R. Tee, J. Tait-Mulder, A. Di Nardo, J. M. Han, E. Kwiatkowski, E. A. Dunlop, K. M. Dodd, R. D. Folkerth, P. L. Faust, M. B. Kastan, M. Sahin and C. L. Walker (2013). "A tuberous sclerosis complex signalling node at the peroxisome regulates mTORC1 and autophagy in response to ROS." Nat Cell Biol **15**(10): 1186-1196.

Zhang, L., J. S. Cardinal, R. Bahar, J. Evankovich, H. Huang, G. Nace, T. R. Billiar, M. R. Rosengart, P. Pan and A. Tsung (2012). "Interferon regulatory factor-1 regulates the autophagic response in LPS-stimulated macrophages through nitric oxide." Mol Med **18**: 201-208.

Zhao, Y., J. Yang, W. Liao, X. Liu, H. Zhang, S. Wang, D. Wang, J. Feng, L. Yu and W. G. Zhu (2010). "Cytosolic FoxO1 is essential for the induction of autophagy and tumour suppressor activity." Nat Cell Biol **12**(7): 665-675.

Zheng, Y. H., C. Tian, Y. Meng, Y. W. Qin, Y. H. Du, J. Du and H. H. Li (2012). "Osteopontin stimulates autophagy via integrin/CD44 and p38 MAPK signaling pathways in vascular smooth muscle cells." J Cell Physiol **227**(1): 127-135.

Zhong, W., H. Zhu, F. Sheng, Y. Tian, J. Zhou, Y. Chen, S. Li and J. Lin (2014). "Activation of the MAPK11/12/13/14 (p38 MAPK) pathway regulates the transcription of autophagy genes in response to oxidative stress induced by a novel copper complex in HeLa cells." Autophagy **10**(7): 1285-1300.

Zhu, H., M. Foretz, Z. Xie, M. Zhang, Z. Zhu, J. Xing, J. Leclerc, M. Gaudry, B. Viollet and M. H. Zou (2014). "PRKAA1/AMPKalpha1 is required for autophagy-dependent mitochondrial clearance during erythrocyte maturation." Autophagy **10**(9): 1522-1534.
