# Supplementary figures and images for: iLIR database: A web resource for LIR motif-containing proteins in eukaryotes
Source: Autophagy. 2016 Aug 2;12(10):1945–53. doi: 10.1080/15548627.2016.1207016 (PMC5079668; doi:10.1080/15548627.2016.1207016)

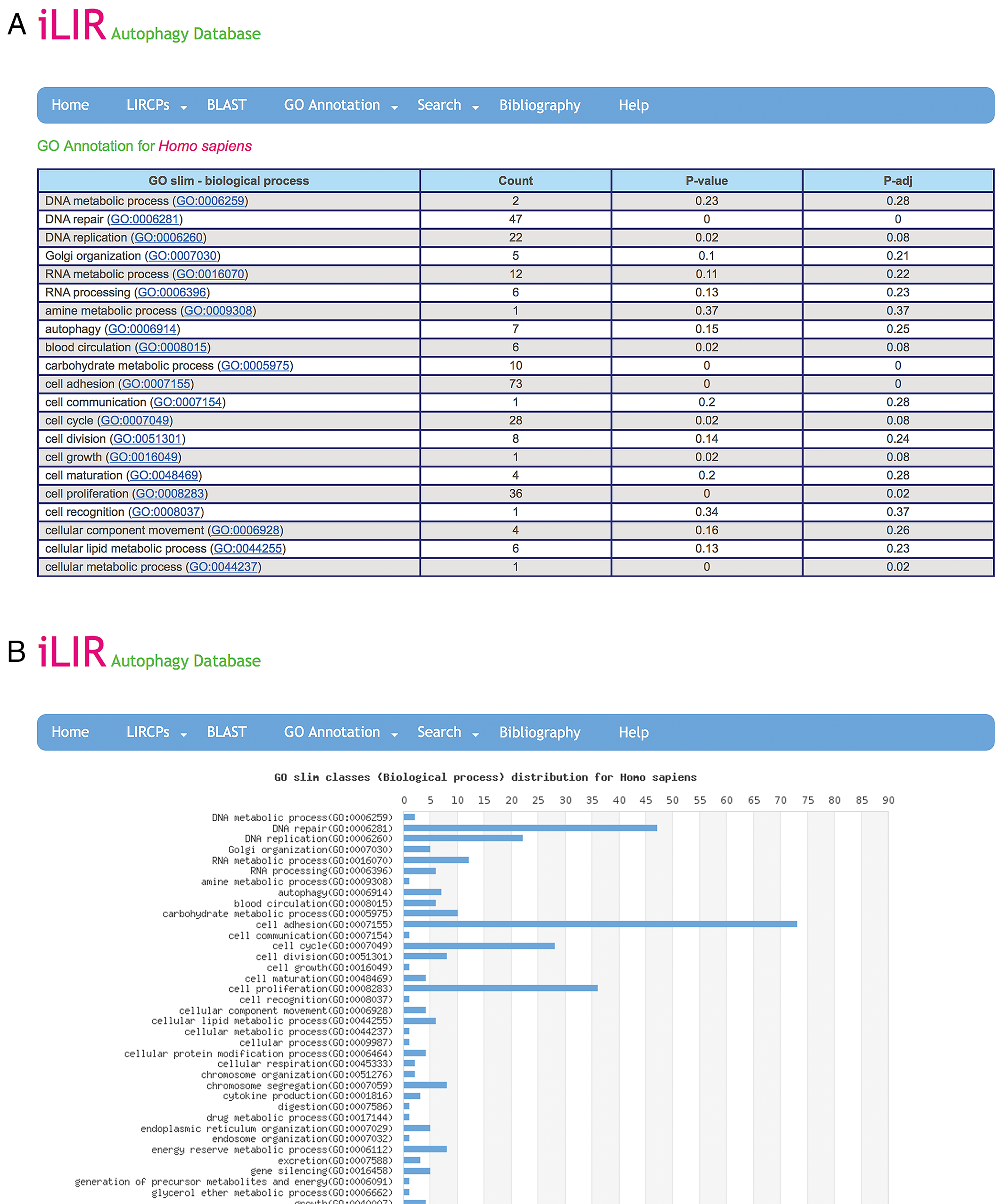

Supplement: KAUP_A_1207016_Supplementary_material.zip [file kaup-12-10-1207016-s001.zip › 2016AUTO0134R2-s03.tif]
